# Lymphadenopathy and fever in a chef during a stay in Europe*

**DOI:** 10.1590/S1806-37132015000004412

**Published:** 2015

**Authors:** Letícia Kawano-Dourado, Daniel Antunes Silva Peirera, Alexandre de Melo Kawassaki, Marisa Dolhnikoff, Marcos Vinicius da Silva, Ronaldo Adib Kairalla

**Affiliations:** University of São Paulo, School of Medicine, Hospital das Clínicas, São Paulo, Brazil. Interstitial Lung Disease Group, Instituto do Coração - InCor, Heart Institute - University of São Paulo School of Medicine Hospital das Clínicas, São Paulo, Brazil; University of São Paulo, School of Medicine, Hospital das Clínicas, São Paulo, Brazil. Interstitial Lung Disease Group, Instituto do Coração - InCor, Heart Institute - University of São Paulo School of Medicine Hospital das Clínicas, São Paulo, Brazil; University of São Paulo, School of Medicine, Hospital das Clínicas, São Paulo, Brazil. Interstitial Lung Disease Group, Instituto do Coração - InCor, Heart Institute - University of São Paulo School of Medicine Hospital das Clínicas, São Paulo, Brazil; University of São Paulo, School of Medicine, Department of Pathology, São Paulo, Brazil. Department of Pathology, University of São Paulo School of Medicine, São Paulo, Brazil; Emílio Ribas Institute of Infectious Diseases, São Paulo, Brazil. Emílio Ribas Institute of Infectious Diseases, São Paulo, Brazil; University of São Paulo, School of Medicine, São Paulo, Brazil. Interstitial Lung Disease Group, Instituto do Coração - InCor, Heart Institute - University of São Paulo School of Medicine Hospital das Clínicas, São Paulo, Brazil; and Professor. Pulmonary Division, Department of Cardiorespiratory Diseases, University of São Paulo School of Medicine, São Paulo, Brazil

**Keywords:** Brucellosis, Fever, Lymph nodes, Brucella, Mononuclear phagocyte system, Granuloma

## Abstract

This case illustrates a rare presentation (as lymphadenopathy and fever) of one of the most common zoonotic diseases worldwide-brucellosis-in a 22-year-old Brazilian male (a chef) who had recently returned to Brazil after having lived in and traveled around Europe for one year. The histopathology, clinical history, and response to treatment were all consistent with a diagnosis of brucellosis, which was confirmed by PCR in a urine sample. We also review some aspects of brucellosis, such as the clinical features, diagnosis, and management.

## Introduction

The case reported here illustrates the differential diagnosis of febrile lymphadenopathy secondary to necrotizing granulomas. Lymphadenopathy and fever secondary to necrotizing granulomas can be a challenging clinical scenario. The differential diagnosis of necrotizing granuloma typically includes, but is not limited to, fungal or mycobacterial infections. Although bacteria such as *Brucella* spp. can cause necrotizing granulomas, they are often overlooked as causes of granulomatous lesions. When such bacteria represent a potential etiologic factor, a negative bacterial culture should be interpreted with caution, given that *Brucella* spp. are fastidious organisms. We also review various aspects of brucellosis, the most common zoonotic disease worldwide.

## Case report

A previously healthy 22-year-old man was referred to our hospital with an 8-week history of cervical and mediastinal lymphadenopathy, fatigue, and intermittent fever. The patient reported no excessive sweating or weight loss. He had been living in Europe (in London) for one year, where he had been working as a chef, when his illness prompted him to return to Brazil. 

**Figure 1 -:**
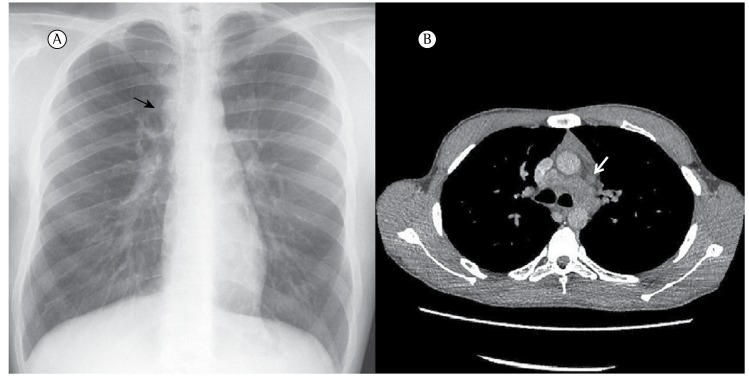
(A) Chest X-ray, posteroanterior view, showing a widened paratracheal stripe (arrow). (B) Highresolution CT of the chest revealing enlarged mediastinal lymph nodes (arrow).

**Figure 2 -:**
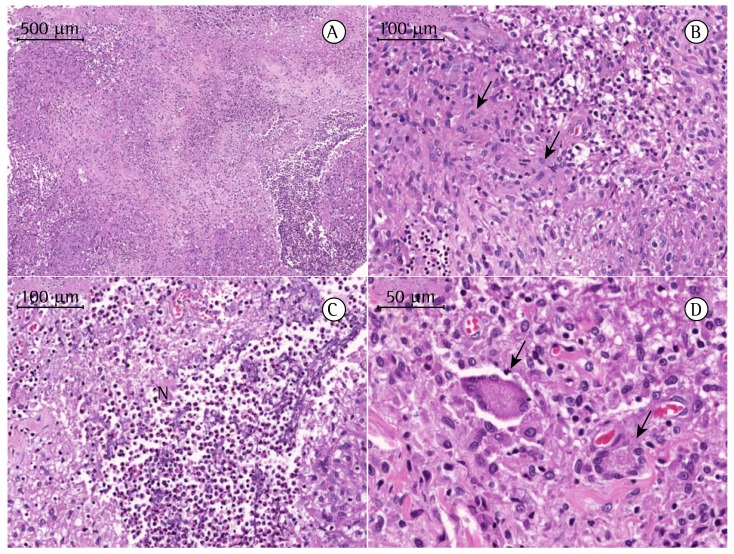
Photomicrographs of a cervical lymph node biopsy sample. Lymphoid tissue is replaced by necrotizing granulomatous inflammation (panel A). Note palisading epithelioid cells (panel B, arrows), extensive neutrophil-rich suppurative necrosis (panel C, letter N), and scattered giant cells (panel D, arrows). Hematoxylin-eosin staining (magnification varies; see the scale bars displayed in the panels).

On physical examination, we observed painless, rubbery bilateral anterior cervical lymph nodes, 2 cm in diameter, and a nonhealing sinus tract at the site of a previous fine needle aspiration that had been performed at another facility and had yielded an inconclusive result. There were no skin or oral lesions, the teeth were in good condition, and the patient had no respiratory complaints. A chest X-ray showed widening of the right paratracheal stripe, and a subsequent CT scan of the chest revealed enlarged mediastinal lymph nodes (Figure 1). The patient had a normal white cell count (6.5 × 10^9^/L), with normal lymphocytes. His liver function was normal; an autoantibody panel was negative; he produced an induration of 20 mm (positive result) in response to a PPD skin test; and a cryptococcal antigen test was negative. In addition, serologic testing for HIV was negative, as were tests for histoplasmosis (immunodiffusion), toxoplasmosis (ELISA), tularemia (agglutination), and cat scratch disease (indirect fluorescence assay). An excisional cervical lymph node biopsy showed a suppurative (neutrophilic), necrotizing granulomatous lesion (Figure 2). However, on direct examination of the specimen, we identified no bacteria (Gram staining), fungi (Grocott methenamine silver staining), or acid-fast bacteria (Ziehl-Neelsen staining). Cultures for bacteria, mycobacteria, and fungi were also negative. 

Despite the 20-mm PPD induration, mycobacterial infection does not typically present an exuberant inflammatory response manifesting as suppurative, necrotizing granulomatous lesions. Although the most common cause of such lesions is fungal infection, there have been reports in which suppurative, necrotizing granulomatous lesions have been attributed to infection with certain bacteria^(^ 1 ^)^: *Francisella tularensis* (tularemia); *Bartonella henselae* (cat scratch disease); *Actinomyces *spp*.*; *Burkholderia pseudomallei* (melioidosis); *Chlamydia trachomatis* (Lymphogranuloma venereum); and *Brucella *spp*.* (brucellosis).

A detailed history regarding exposures was taken. As a chef, the patient had a variety of gastronomic experiences during his stay in London and his travels around Europe (to Eastern Europe, Portugal, and Spain). He reported exposure to unpasteurized sheep cheese and to exotic raw meats. Therefore, we considered brucellosis a possible differential diagnosis. Because the bacterial culture results were negative, we employed PCR as an alternative method to reach the diagnosis. We performed PCR using primers targeting the *bcsp31* gene sequence for *Brucella *spp. (the B4/B5 primer pair) in urine. The PCR was positive for *Brucella *spp., which allowed us to confirm the suspected diagnosis of brucellosis, given that the histopathology, clinical history, and response to treatment (mentioned further on) were all consistent with that diagnosis.

## Discussion

Brucellosis is a chronic granulomatous zoonosis caused by intracellular bacteria of the genus *Brucella*. It is transmitted to humans through contact with fluids from infected animals (especially through the consumption of mutton and beef, as well as of the milk of sheep and cows) or through direct contact with infected animal parts (such as the placenta, by inoculation through ruptures of skin and mucous membranes) or even by inhalation of aerosolized infectious particles. ^(^ 2 ^)^ Consumption of unpasteurized dairy products is the most common means of transmission.^(^ 2 ^,^ 3 ^)^

Human brucellosis is one of the most common zoonotic diseases worldwide. Although its epidemiology has drastically changed over the past decades and control of the disease has been achieved in a number of areas where it was traditionally endemic, the Mediterranean basin (around which our patient had been traveling) continues to be recognized as a region in which brucellosis is endemic.^(^ 3 ^)^

The wide spectrum of clinical manifestations of human brucellosis has earned it a place alongside syphilis and tuberculosis as one of the "great imitators". In patients with brucellosis, practically every organ and system of the human body can be affected. The physical examination findings are generally nonspecific, although lymphadenopathy, hepatomegaly, or splenomegaly is often present due to the tropism of *Brucella *spp. for the reticuloendothelial system. In addition, isolated lymphadenopathy is rare in human brucellosis. Because of the protean clinical manifestations, the cornerstone of making the clinical diagnosis of brucellosis is taking a detailed history and paying careful attention to epidemiological information. Special attention must also be paid to determining whether the patient has ingested contaminated dairy products or has been in contact with infected animals. Detailed patient interviews are crucial to making the diagnosis of human brucellosis, especially in urban and non-endemic areas, as well as when travelers acquire the disease abroad and become ill in non-endemic settings.^(^ 3 ^,^ 4 ^)^

The gold standard for the diagnosis of brucellosis is isolation of the bacteria from blood or tissue samples. Making a diagnosis of brucellosis can be quite challenging, because blood cultures or cultures of the tissue fragment are positive in only 15-70% of cases, as well as because the detection of *Brucella *spp. requires a prolonged incubation time.^(^ 5 ^)^ Bone marrow cultures can increase the sensitivity by 15-20% over that of blood cultures.^(^ 4 ^)^ However, in many cases, clinicians must use a wide range of nonspecific routine hematological and biochemical tests, together with *Brucella*-specific assays (serological and molecular techniques), in order to reach a definitive diagnosis.^(^ 6 ^,^ 7 ^)^ Each of those tests has advantages and limitations, therefore requiring careful interpretation of the results. Serological assays, which are mainly based on the identification of lipopolysaccharide antigens of *Brucella*, have high sensitivity but low specificity (as low as 64% in some reports), due to cross-reactivity with other bacterial species.^(^ 4 ^,^ 8 ^)^ The fact that antibodies can be detectable for months after therapy further complicates the use of serological assays for the identification of relapse and reinfection. Nevertheless, a number of serological methods can be useful. One such method is serum agglutination testing, the modality for which there is the greatest amount of data in the literature. In an appropriate clinical scenario, a fourfold or greater increase in the *Brucella* agglutination titer between acute- and convalescent-phase serum samples, obtained ≥ 2 weeks apart, confirms a diagnosis of brucellosis. Absolute thresholds for serum agglutination testing should be individualized: positivity is defined as a titer of 1:160-1:320 in endemic regions and as a titer of 1:80 in non-endemic regions.^(^ 9 ^)^ Other methods, such as ELISA, the direct antiglobulin (Coombs) test, and the immunocapture test, are available but do not seem to overcome the aforementioned problems.^(^ 9 ^)^

Another technique that has been increasingly used in the diagnosis of brucellosis is PCR. PCR is not a routine diagnostic method but can be performed on any clinical specimen and has been shown to have excellent sensitivity and specificity.^(^ 7 ^,^ 8 ^,^ 10 ^-^ 12 ^)^ Genus-specific PCR targeting *bcsp31* seems to have greater sensitivity than do those targeting any other *Brucella* gene sequence available.^(^ 7 ^)^ The sensitivity and specificity of PCR assays are both between 90% and 100%.^(^ 8 ^,^ 12 ^)^ As in other infectious diseases, PCR testing is becoming an excellent alternative method for diagnosing brucellosis when standard methods have failed or are not available, especially when the clinical and histopathological aspects are consistent with the diagnosis.^(^ 9 ^,^ 13 ^-^ 15 ^)^ A growing body of evidence indicates that PCR assays are accurate methods for the diagnosis of brucellosis, although there is a need for standardization before their widespread use as such can be recommended.

The goal of brucellosis treatment is the resolution of infection and the prevention of complications, relapses, and sequelae. The optimal treatment of uncomplicated brucellosis (without spondylitis, neurobrucellosis, or endocarditis) is based on a 6-week regimen of doxycycline, combined with streptomycin for 2-3 weeks or with rifampin for 6 weeks.^(^ 16 ^)^ Although the streptomycin-containing regimen is slightly more efficacious in preventing relapse, parenteral administration of streptomycin complicates its use, and the doxycycline-rifampin regimen is therefore used more frequently, because of its convenience.^(^ 17 ^,^ 18 ^)^ A 6-week regimen of quinolone plus rifampin is slightly more well tolerated than is that of doxycycline plus rifampin, and low quality evidence did not show any difference in overall effectiveness.^(^ 19 ^)^ There is also some evidence that a three-drug regimen (involving the addition of trimethoprim-sulfamethoxazole to either of the abovementioned two-drug regimens, or a combination of streptomycin, rifampin and doxycycline) is an effective therapy in complex cases. Extended treatment (for at least 12 weeks) and the use of three-drug regimens should be considered in patients with complicated disease.^(^ 20 ^)^

In the case presented here, the patient was treated with doxycycline and rifampin. After 6 weeks, he presented complete resolution of fatigue and lymphadenopathy. At this writing, the patient has been followed for two years after the completion of treatment and there has been no evidence of relapse.

Our case illustrates a rare presentation of brucellosis, one of the most common zoonotic diseases worldwide. It also highlights the importance of taking a detailed epidemiological history as an important tool to guide clinicians to a correct diagnosis of infectious granulomatous diseases such as brucellosis.

## References

[1] El-Zammar OA, Katzenstein AL (2007). Pathological diagnosis of granulomatous lung disease: a review. Histopathology.

[2] Bosilkovski M, Krteva L, Dimzova M, Vidinic I, Sopova Z, Spasovska K (2010). Human brucellosis in Macedonia - 10 years of clinical experience in endemic region. Croat Med J.

[3] Pappas G, Papadimitriou P, Akritidis N, Christou L, Tsianos EV (2006). The new global map of human brucellosis. Lancet Infect Dis.

[4] Franco MP, Mulder M, Gilman RH, Smits HL (2007). Human brucellosis. Lancet Infect Dis.

[5] Memish Z, Mah MW, Al Mahmoud S, Al Shaalan M, Khan MY (2000). Brucella bacteraemia: clinical and laboratory observations in 160 patients. J Infect.

[6] Araj GF (2010). Update on laboratory diagnosis of human brucellosis. Int J Antimicrob Agents.

[7] Yu WL, Nielsen K (2010). Review of detection of Brucella spp: by polymerase chain reaction. Croat Med J.

[8] Sohrabi M, Mohabati Mobarez A, Khoramabadi N, Hosseini Doust R, Behmanesh M (2014). Efficient Diagnosis and Treatment Follow-up of Human Brucellosis by a Novel Quantitative TaqMan Real-Time PCR assay: a Human Clinical Survey. J Clin Microbiol.

[9] Christopher S, Umapathy BL, Ravikumar KL (2010). Brucellosis: review on the recent trends in pathogenicity and laboratory diagnosis. J Lab Physicians.

[10] Queipo-Ortu-o MI, Colmenero JD, Mu-oz N, Baeza G, Clavijo E, Morata P (2006). Rapid diagnosis of Brucella epididymo-orchitis by real-time polymerase chain reaction assay in urine samples. J Urol.

[11] Baddour MM, Alkhalifa DH (2008). Evaluation of three polymerase chain reaction techniques for detection of Brucella DNA in peripheral human blood. Can J Microbiol.

[12] Sanjuan-Jimenez R, Morata P, Bermúdez P, Bravo MJ, Colmenero JD (2013). Comparative clinical study of different multiplex real time PCR strategies for the simultaneous differential diagnosis between extrapulmonary tuberculosis and focal complications of brucellosis. PLoS Negl Trop Dis.

[13] Mitka S, Anetakis C, Souliou E, Diza E, Kansouzidou A (2007). Evaluation of different PCR assays for early detection of acute and relapsing brucellosis in humans in comparison with conventional methods. J Clin Microbiol.

[14] Nimri LF (2003). Diagnosis of recent and relapsed cases of human brucellosis by PCR assay. BMC Infect Dis.

[15] Morata P, Queipo-Ortu-o MI, Reguera JM, García-Ordo-ez MA, Cárdenas A, Colmenero JD (2003). Development and evaluation of a PCR-enzyme-linked immunosorbent assay for diagnosis of human brucellosis. J Clin Microbiol.

[16] Ariza J, Bosilkovski M, Cascio A, Colmenero JD, Corbel MJ, Falagas ME (2007). Perspectives for the treatment of brucellosis in the 21st century: the Ioannina recommendations. PLoS Med.

[17] Pappas G, Siozopoulou V, Akritidis N, Falagas ME (2007). Doxycycline-rifampicin: physicians' inferior choice in brucellosis or how convenience reigns over science. J Infect.

[18] Solís García del Pozo J, Solera J (2012). Systematic review and meta-analysis of randomized clinical trials in the treatment of human brucellosis. PLoS One.

[19] Yousefi-Nooraie R, Mortaz-Hejri S, Mehrani M, Sadeghipour P (2012). Antibiotics for treating human brucellosis. Cochrane Database Syst Rev.

[20] Mantur BG, Amarnath SK, Shinde RS (2007). Review of clinical and laboratory features of human brucellosis. Indian J Med Microbiol.

